# Bromide of Sodium

**Published:** 1871-12

**Authors:** 


BROMIDE OF SODIUM.—As it is generally acknowledged that the prolonged use of bromide of potassium, in epilepsy and other nervous disorders, is often attended with serious inconveniences, and even dangers, Dr. Meredith Clymer, of New York, (*Med. World*), believes that in the *bromide of sodium* a happy substitute has been found that will fully meet every indication for which the bromide of potassium has been given, and is free from the objections which are justly urged against the latter. The taste of the bromide of sodium is much less unpleasant than that of the bromide of potassium, being very like common salt, and it may be used to replace the latter, mixed with the food, as with bread, butter, eggs, in milk, etc. Hence it is of more easy administration than bromide of potassium, to the taste of which some persons have invincible repugnance, and increasing with its use. It is of the first importance that bromide of sodium should be perfectly free of all impurities, particularly of iodine. Larger doses of the hydrated salt are required than of the anhydrous, for it crystallizes with four equivalents of water.

The doses of bromide of sodium are about the same as those of bromide of potassium. In epilepsy he usually gives 20 grains three times daily, and has rarely gone above that amount. It sometimes seems to cause or encourage constipation.

